# Epidemio-toxicological profile of suicide cases: analysis from a forensic unit in Brazil

**DOI:** 10.1080/20961790.2022.2113622

**Published:** 2023-02-12

**Authors:** Carolina de Castro Martins, Yara Viera Lemos, Maycoln Leoni Martins Teodoro, Ana Paula Drummond-Lage

**Affiliations:** a Department of Phsycology, Universidade Federal de Minas Gerais, Belo Horizonte, MG, Brazil; b Department of Forensic Anthropology, Polícia Civil de Minas Gerais, Belo Horizonte, MG, Brazil; c School of Medicine, Faculdade de Ciências Médicas de Minas Gerais, Belo Horizonte, MG, Brazil; d Post-Graduation Department, Faculdade de Ciências Médicas de Minas Gerais, Belo Horizonte, MG, Brazil

**Keywords:** Forensic sciences, forensic toxicology, forensic medicine, forensic anthropology, suicide

## Abstract

The suicide phenomenon involves complex interactions between psychological, biological, cultural and socio-environmental factors. This study aimed to assess the epidemiological and toxicological profiles of officially confirmed suicide victims. A retrospective study was performed using autopsy reports, forensic anthropology examinations and police summaries of all suicide cases that underwent toxicological analysis at an Official Forensic Laboratory (Minas Gerais, Brazil) in 1 year. The sample set was composed of 351 suicide victims, predominantly men (70.32%), most of them were adults between 31 and 64 years old (62.11%), with mixed skin colour (48.89%) and low educational level (66.44%). The most common suicide method was hanging (57.79%), followed by intoxication (30.45%). Most victims presented positive toxicological results (56.41%), especially for the presence of medicines (37.6%), illicit drugs (36.3%) and pesticides (26.1%). Our study corroborated previous data that most suicide victims have low educational levels. The most common toxicological findings were medicines, especially prescription drugs, followed by illicit drugs and pesticides. We hope this study contributes to reflections and planning of preventive suicide programmes, considering the described profiles of victims.

## Key points

Suicide is a global phenomenon. Each year, more than 793 000 suicides occur worldwide, which result from interactions between psychological, biological, cultural and socio-environmental factors.

The goal of this study was to describe and analyze the epidemio-toxicological profiles of 351 suicide victims in a Forensic Unit in Southwest Brazil.

The victims were mainly men (70.32%), most of the adults between 31 and 64 years old (62.11%), with mixed skin colour (48.89%) and low educational level (66.44%).

The most common suicide method was hanging (57.79%), followed by intoxication (30.45%). Most victims presented positive toxicological results (56.41%), especially for the presence of medicines (37.6%), illicit drugs (36.3%) and pesticides (26.1%).

## Introduction

Suicide is a global phenomenon that can be understood as a result of complex interactions between psychological, biological, cultural and socio-environmental factors [1,2]. Every year, more than 793 000 people die worldwide from suicide, and for each suicide death, attempted suicide is estimated to be 20 times more frequent [3].

An increased risk of committing suicide is associated with factors such as the presence of a psychiatric disorder, consumption of illicit drugs and substance abuse and previous suicide attempts. Suicide is also possibly related to unemployment, low educational levels and high socioeconomic inequality [3–5]. Several authors suggest that the prevalence of different suicide methods varies according to the population's culture, availability and acceptability [6–8].

Some studies have shown a significant increase in suicide rates in Brazil since 1980 [5, 9–11]. The primary methods are hanging, firearm injury and self-poisoning with pesticides [12,13]. Currently, Brazil is the country with the ninth highest absolute number of deaths from suicide. More than 11 000 suicide deaths are registered every year in Brazil, and it is the fourth most common cause of death in adolescents and young adults from 15 to 29 years old [6, 9, 14].

However, it is likely that these statistics are underestimated. Because determining the manner of death to be suicide involves intent determination, suicide deaths may be underreported and mistakenly classified as accidents or deaths from other causes [15]. In Brazil, mandatory notification of public authorities is required in the case of a violent or suspicious death, including suicide. The victim’s body must undergo a standardized autopsy by a forensic expert doctor, including a toxicological investigation.

The Legal Medical Institute from Belo Horizonte (IML-BH) is responsible for toxicological investigations of violent and suspicious deaths across Minas Gerais State, the second most populous and fourth largest by area that accounts for the third-largest gross domestic product (GDP) of all 26 Brazilian states [16,17].

Here, we aimed to determine the relationships between suicide methods and illicit drugs, medicines and pesticides assessed through the epidemiological and toxicological profiles of suicide victims from Minas Gerais.

## Methods

A retrospective study was performed using autopsy reports, forensic anthropology examinations and police summaries of all suicide cases that underwent toxicological analysis at IML-BH within 1 year. Toxicological research was performed by the Forensic Laboratory of Toxicology (LabTox), which is the main forensic toxicology centre in Minas Gerais, responsible for more than 12 000 toxicological analyses every year [18]. All confirmed cases of death by suicide after the end of the police investigations were included.

Additional information about death circumstances was collected from the Integrated System of Social Defense of Minas Gerais, a police force integrated database system. The data are available through the Record Events of Social Defense system [19]. For toxicological examinations, analysis was performed on blood, urine or viscera samples by validated analytical methods, such as thin-layer chromatography (TLC), high-performance liquid chromatography (HPLC), gas chromatography-mass spectrometry (GC-MS) and immunochromatographic technique (ICT). A screening test for the presence of cannabis (as determined by the presence of δ-9-tetrahydrocannabinol (THC) and/or THC acid), morphine, methadone, methamphetamine, cocaine (as determined by the presence of cocaine itself and/or the presence of metabolites of cocaine), 3,4-methylenedioxymethamphetamine (MDMA), benzodiazepines, antidepressants and antipsychotic medications was performed for all cases. The presence of an illicit substance was defined by a positive result for any of the following: cannabis, methamphetamine, cocaine or MDMA.

Variables collected were stratified in sociodemographic (sex, age, marital status, schooling, skin colour and suicide method) and toxicological data. Depending on the state of decomposition and taphonomic factors, sex and age were determined by a multidisciplinary team of forensic anthropologists. In those cases, the skin colour was determined only after identification and using data from official documents. The main methods used to access data of interest regarding the biological profile were probabilistic sex diagnosis (DSP) [20] and two step procedure (TSP) [21].

For statistical analysis, the age was classified according to World Health Organization (WHO) criteria: child (1 to 9 years old), adolescent (10 to 19 years old), young adult (20 to 30 years old), adult (31 to 64 years old) and elderly (65 years old or older) [22]. The marital status included two categories: couple (married or living with a partner) and single (widowed, separated, divorced or single). Results were expressed in frequencies and percentages for the categorical variables and with average, minimum and maximum values for the continued quantitative variables. The association between variables was tested with Chi-square tests and Fisher exact tests. Loglinear modelling analysis was used to determine multivariate associations. Odds ratios (ORs) are reported as a measure of the association between sociodemographic variables and suicide methods. The results were considered significant when *P* < 0.05. The analyses were performed using the Stata Version 15 (StataCorp LLC, College Station, TX, USA).

## Results

Overall, 351 forensic toxicological analyses of suicide cases were required and conducted by LabTox in 1 year. Sociodemographic characteristics and toxicological results of all 351 suicide victims showed that 70.32% were male and 48.89% had a mixed skin colour. In 81 cases (23.07%), it was not possible to retrieve data about skin colour because no official documents were available. The suicide victims were aged 13 to 92 years (40.72 ± 14.61) and 62.11% were adults between 31 and 64 years old. Hanging was the most common method (57.79%), followed by exogenous intoxication (30.45%). Most victims did not have a partner (54.59%) according to the official documents (Table 1).

**Table 1.** Characteristics of suicide victims and toxicological results.

	Results of the toxicological analysis		Total (*n* = 351)
Variables	Negative (*n* = 153)	Positive (*n* = 198)	
Sex*	*P* = 0.028^a^		
Male	114 (76.51%)	130 (65.66%)	244 (70.32%)
Female	35 (23.49%)	68 (34.34%)	103 (29.68%)
Age (years)	*P* = 0.168^b^		
10–19	8 (5.23%)	6 (3.03%)	14 (3.98%)
20–30	37 (24.18%)	42 (21.21%)	79 (22.51%)
31–64	86 (56.21%)	132 (66.67%)	218 (62.11%)
≥65	22 (14.37%)	18 (9.09%)	40 (11.39%)
Marital status*	*P* = 0.268^a^		
Single	48 (50.53%)	59 (58.42%)	107 (54.59%)
Couple	47 (49.47%)	42 (41.58%)	89 (45.41%)
Schooling*	*P* = 0.798^b^		
Less than high school	44 (63.77%)	55 (68.75%)	99 (66.44%)
High school	17 (24.64%)	16 (20.00%)	33 (22.15%)
More than high school	8 (11.59%)	9 (11.25%)	17 (11.41%)
Skin colour*	*P* = 0.001^b^		
White	63 (52.94%)	46 (30.46%)	109 (40.37%)
Mixed	44 (36.98%)	88 (58.28%)	132 (48.89%)
Black	12 (10.08%)	17 (11.26%)	29 (10.74%)
Method*	*P* ≤ 0.000^a^		
Hanging	104 (78.79%)	63 (40.13%)	167 (57.79%)
Exogenous intoxication	6 (4.55%)	82 (52.23%)	88 (30.45%)
Fall from high height	11 (8.33%)	4 (2.55%)	15 (5.19%)
Fire gun	8 (6.06%)	6 (3.82%)	14 (4.84%)
Other**	3 (2.27%)	2 (1.27%)	5 (1.73%)

* Missing variables.

** The category “other” includes thermal injuries (*n* = 2) and sharp force injury (*n* = 3).

a The *P*-values refer to the tests chi-square of independence.

b The *P*-values refer to the tests Fisher exact test.

The toxicological examinations indicated that 56.41% of the victims presented positive results for an illicit substance. Both males and females displayed more positive results than negatives (*P* = 0.028). Negative results were more common for white skin colour victims (52.94%), while mixed skin colour victims had more positive results (58.28%) (*P* = 0.001) (Table 1).

Table 2 shows the associations between sociodemographic characteristics and toxicological findings according to the suicide method. After assessing hanging cases, a higher number of negative toxicological examination results were observed for adult victims (age 31 to 64; 61.54%) (*P* = 0.000) and positive results for young adults from 20 to 30 years old (39.68%). Skin colour was also associated with hanging: mixed skin colour victims used this method more than white skin colour victims (69.09% *vs*. 23.64%; *P* = 0.001). There was an association between positive intoxication results and adult victims (74.39%; *P* = 0.081). For other suicide methods, no significant association was found between sociodemographic characteristics and toxicological findings.

**Table 2.** Toxicological results according to suicide methods *vs*. sociodemographic characteristics.

	Hanging		Intoxication		Fall from high height		Fire gun		Other**	
	Negative (*n* = 104)	Positive (*n* = 63)	Negative (*n* = 6)	Positive (*n* = 82)	Negative (*n* = 11)	Positive (*n* = 4)	Negative (*n* = 8)	Positive (*n* = 6)	Negative (*n* = 3)	Positive (*n* = 2)
Sex*	*P* = 0.369		*P* = 1.000		*P* = 1.000		*P* = 0.580		*P* = 1.000	
Male	84 (83.17%)	56 (88.89%)	3 (50.00%)	45 (54.88%)	7 (63.64%)	3 (75.00%)	6 (75.00%)	3 (50.00%)	2 (66.67%)	2 (100%)
Female	17 (16.83%)	7 (11.11%)	3 (50.00%)	37 (45.12%)	4 (36.36%)	1 (25.00%)	2 (25.00%)	3 (50.00%)	1 (33.33%)	0 (0%)
Age (years)	*P* ≤ 0.000		*P* = 0.081		*P* = 0.549		*P* = 0.748		*P* = 1.000	
10–19	5 (4.81%)	1 (1.59%)	1 (16.67%)	3 (3.66%)	1 (9.09%)	1 (25.00%)	0 (0%)	0 (0%)	0 (0%)	0 (0%)
20–30	20 (19.23%)	25 (39.68%)	2 (33.33%)	11 (13.41%)	4 (36.36%)	0 (0%)	2 (25.00%)	2 (33.33%)	1 (33.33%)	0 (0%)
31–64	64 (61.54%)	37 (58.73%)	2 (33.33%)	61 (74.39%)	5 (45.46%)	3 (75.00%)	6 (75.00%)	3 (50.00%)	2 (66.67%)	2 (100%)
≥65	15 (14.42%)	0 (0%)	1 (16.67%)	7 (8.54%)	1 (9.09%)	0 (0%)	0 (0%)	1 (16.67%)	0 (0%)	0 (0%)
Marital status*	*P* = 0.237		*P* = 1.000		*P* = 1.000		*P* = 0.524		*P* = 1.000	
Single	37 (52.11%)	25 (64.10%)	2 (50.00%)	23 (52.27%)	4 (66.67%)	3 (75.00%)	2 (33.33%)	2 (66.67%)	1 (100%)	1 (100%)
Couple	34 (47.89%)	14 (35.90%)	2 (50.00%)	21 (47.73%)	2 (33.33%)	1 (25.00%)	4 (66.67%)	1 (33.33%)	0 (0%)	0 (0%)
Schooling*	*P* = 0.717		*P* = 0.31		*P* = 1.000		*P* = 0.679		*P* = 1.000	
Less than high school	38 (73.10%)	19 (73.08%)	0 (0%)	29 (70.73%)	1 (25.00%)	1 (50.00%)	1 (20.00%)	2 (66.67%)	1 (100%)	0 (0%)
High school	9 (17.31%)	6 (23.08%)	0 (0%)	6 (14.63%)	2 (50.00%)	0 (0%)	3 (60.00%)	1 (33.33%)	0 (0%)	1 (100%)
More than high school	5 (9.61%)	1 (3.84%)	1 (100%)	6 (14.63%)	1 (25.00%)	1 (50.00%)	1 (20.00%)	0 (0%)	0 (0%)	0 (0%)
Skin colour*	*P* = 0.001		*P* = 1.000		*P* = 1.000		*P* = 0.033		*P* = 1.000	
White	44 (51.77%)	13 (23.64%)	1 (33.33%)	24 (34.29%)	5 (55.56%)	2 (66.67%)	6 (85.71%)	0 (0%)	1 (50.00%)	1 (100%)
Mixed	33 (38.82%)	38 (69.09%)	2 (66.67%)	36 (51.43%)	3 (33.33%)	1 (33.33%)	1 (14.29%)	2 (66.67%)	1 (50.00%)	0 (0%)
Black	8 (9.41%)	4 (7.27%)	0 (0%)	10 (14.28%)	1 (11.11%)	0 (0%)	0 (0%)	1 (33.33%)	0 (0%)	0 (0%)

* Missing variables.

** The category “other” includes thermal injuries (*n* = 2) and sharp force injury (*n* = 3).

Number are rounded so the percentages may not add up to 100%.

In-depth investigations detailed the relationships between suicide methods and toxicological results. All positive results (198 cases) are presented in Table 3. The data suggest that the most common toxicological findings were medicines (37.6%), followed by illicit drugs (36.3%) and pesticides (26.1%). Of those who hanged themselves (*n* = 63), 76.2% were positive for illicit drugs, mainly cocaine and its derivatives (93.0%). For exogenous intoxication cases (*n* = 82), pesticides accounted for 48.8% of positive cases, followed by medicines (47.6%). The most frequent medicines were anticonvulsants, barbiturates, sedatives, and hypnotics (45.6%) and antidepressants (41.0%).

**Table 3.** Substance distribution in toxicological findings and suicide methods (*N* = 157).

Toxicological findings	Total	Hanging	Intoxication	Fall from high height	Fire gun	Other*
Medicines	59 (37.6%)	14 (22.2%)	39 (47.6%)	1 (25.0%)	3 (50.0%)	2 (100%)
Prescription drugs	53 (89.8%)	13 (92.9%)	36 (92.3%)	1 (100%)	3 (100%)	–
Over-the-counter drugs	14 (23.7%)	1 (7.1%)	13 (33.3%)	–	–	–
Anticonvulsants, barbiturates, sedatives and hypnotics	21 (35.6%)	7 (50.0%)	17 (43.6%)	1 (100%)	2 (66.6%)	–
Antidepressants	22 (37.3%)	3 (21.4%)	16 (41.0%)	1 (100%)	2 (66.6%)	–
Benzodiazepines	12 (20.3%)	4 (28.6%)	8 (20.5%)	–	–	–
Analgesics	9 (15.3%)	–	9 (23.1%)	–	–	–
Anaesthetics	6 (10.2%)	1 (7.1%)	5 (12.8%)	–	–	–
Opioids	3 (5.1%)	–	3 (7.7%)	–	–	–
Antipsychotics	3 (5.1%)	–	3 (7.7%)	–	–	–
Antiallergics	3 (5.1%)	1 (7.1%)	2 (5.1%)	–	–	–
Antiparasitic	1 (1.7%)	–	1 (2.6%)	–	–	–
Mood stabilizers	1 (1.7%)	–	1 (2.6%)	–	–	–
Illicit Drugs	57 (36.3%)	48 (76.2%)	3 (3.6%)	3 (75.0%)	3 (50.0%)	–
Cocaine and derivatives	53 (93.0%)	46 (95.8%)	2 (66.7%)	2 (66.7%)	3 (100%)	–
Cannabis	13 (22.8%)	10 (20.8%)	1 (33.3%)	1 (33.3%)	1 (33.3%)	–
Pesticides	41 (26.1%)	1 (1.6%)	40 (48.8%)	–	–	–
Total	157	63	82	4	6	2

Notes: The same individual may present more than one finding in the toxicology examination.

* The category “others” includes thermal injuries (*n* = 2) and sharp force injury (*n* = 3).

Number are rounded so the percentages may not add up to 100%.

The correlations between sociodemographic variables and suicide methods are shown in Table 4. For suicide methods, the higher the person’s age, the less likely the individual was to choose hanging (2% less likely for each year older) (OR = 0.980, *P* < 0.05) and the higher the probability of exogenous intoxication (3.3%) (OR = 1.033*, P* < 0.01) for both sexes. Women were 75.7% less likely to use hanging as a method (OR = 0.243, *P* < 0.001), with exogenous intoxication being the predominant method among women (38.8%). Exogenous intoxication was 4.5 times more likely in females than in males (OR = 4.564; *P* < 0.00) (Table 4).

**Table 4.** Suicide method determinants (*N* = 238).

Variables	Hanging		Exogenous intoxication		Others	
	OR	*P*-value	OR	*P*-value	OR	*P*-value
Female (male defined as a comparison group)	0.243*	0.000	4.564*	0.000	1.146	0.777
Age	0.980**	0.041	1.033*	0.001	0.982	0.239
Constant	4.477*	0.001	0.093*	0.000	0.145*	0.005
Pseudo *R*²	0.0805		0.1142		0.0357	

* significant at 0.01%.

** significant at 0.05%.

Furthermore, women were 2.11 times more likely than men to have a positive result for medicines (OR = 2.110, *P* < 0.05) and have 69.6% fewer chances of a positive result for illicit drugs when compared with men (OR = 0.304, *P* < 0.01). Age also played a role in illicit drug results. Older victims had lower chances of a positive result for drugs (7.5% for each additional year of age; OR = 0.925, *P* < 0.00). Women were approximately 85% more likely to have positive results for pesticides than men (OR = 1.847, *P* > 0.05). Older individuals had higher chances of a positive result for pesticides in both sexes (4% for every one year) (OR = 1.041, *P* < 0.00) (Table 5).

**Table 5.** Toxicological results and substances determinants (*N* = 270).

Variables	Any substance		Medicines		Illicit drugs		Pesticides	
	OR	*P*-value	OR	*P*-value	OR	*P*-value	OR	*P*-value
Female (male defined as comparison group)	1.431	0.217	2.110**	0.018	0.304*	0.007	1.847	0.083
Age	0.996	0.724	1.006	0.476	0.925*	0.000	1.041*	0.000
Constant	1.948	0.088	0.167*	0.000	7.807*	0.001	0.042*	0.000
Pseudo *R*²	0.043		0.021		0.1779		0.0741	

* significant at 0.01.

** significant at 0.05.

## Discussion

This study aimed to determine the relationships between suicide methods and illicit drugs, medicines and pesticides through epidemiological and toxicological profile analyses of all suicide victims from Minas Gerais who underwent 1 year of toxicological examination. A multidisciplinary forensic team was necessary to minimize missing data. Forensic anthropology was fundamental for retrieving sex and age data, especially in cases with expressive taphonomic factors, burned remains or decomposition, by applying the DSP method [20] and TSP procedure [21].

For the sociodemographic characteristics of these samples, 70.3% of the victims were male. Historically, suicide has been more common among men than women, primarily because of differences in method choice, with women using less lethal methods when attempting suicide, as drug/poison ingestion [23–26].

The average age was 40 years, and more than 50% of the samples were adults between 31 and 64 years old, in accordance with literature data [9]. Given that suicide is the third most common cause of death among young people from 15 to 29 years old [27,28], it is essential to note that adolescents and young adults represented 26.7% of our cases. However, there has been accelerated growth in suicide rates among the elderly population worldwide [6], and factors such as depression, social isolation, loss of meaning of life, loss of children and spouses, and inactivity between others are associated with suicide mortality in this age group [27–29]. In our samples, 11.39% (40/351) of the victims were 65 years old or older. Summarizing the elderly group also demands attention as a risk group [23, 25].

For skin colour, 48.49% of suicide victims had mixed skin colour, while 40.37% were white. Brazilian studies have shown a prevalence of mixed skin colour victims in suicide deaths [27], which can be related to the country's skin colour distribution: 45.22% white, 45.06% mixed, 8.86% black, 0.47% yellow and 0.38% indigenous [16, 30]. Our findings also demonstrated that most of the victims were divorced, single or widowed. Individuals living alone with no close relatives have a significantly higher suicide rate [6]. Additionally, family members can be an essential resource in identifying risk factors and searching for help [31]. Some studies have indicated that most suicide victims have a low educational level (less than 8 years of school) [23, 32,33]. Our study corroborated these data, with 66.44% of the cases here having less than a high school degree, in contrast to 11.41% that had more than a high school level education.

Examining the causes of death in these cases was performed using the autopsy reports. Especially in instances of burnt remains and decomposition, anthropological reports provided important contributions to the analysis of bone trauma. The prevalence of suicide methods varies according to the culture and availability of the method itself [24]. A method’s lethality is a decisive risk factor for completed suicide [8, 34]. In our study, we found hanging as the most common method and the most prevalent among men. Intoxication was predominant among women. Our findings corroborate the hypothesis that men tend to choose more lethal methods such as hanging, firearms and falling from high heights, while women are more likely to use medicines and other substances [35]. We also found an effect of age: the older the victim, the lower the chance of them choosing hanging as a suicide method. This finding is consistent with previous research that indicates that the risk of suicide by hanging decreases with age [36], while the risk of suicide with firearms increases [37]. It was impossible to verify this last hypothesis, given the small number of victims who chose this method in our sample set. Controlled firearm commerce can explain this small number of victims [38].

Toxicological results in retrospective studies have provided insights into the frequency of certain types of substances being associated with suicide [39]. In our data, 56.41% of samples had a positive result for any substance: 37.6% positive for medicines and 36.3% for illicit drugs. Drug misuse and abuse are major health problems [40] and about 5% of the world’s adult population are estimated to have used an illicit drug at least once [41]. There is a strong association between substance use disorder and suicide outcomes. Use of illicit drugs, in general, can be considered significant predictors of suicide [42,43]. The investigation of the relationship between suicide methods and toxicological findings showed interesting results. For instance, among those victims with positive toxicological results who hanged themselves, one of the most lethal methods of suicide usually associated with impulsivity [44], 76.2% had positive results for illicit drugs.

Hanging is one of the most lethal suicide methods. Theoretically, almost any material can be used to carry it out [44,45]. In this study, 26.1% of cases had positive results for pesticides. Suicide by pesticide poisoning is the third most critical suicide cause in Brazil, showing an increased prevalence of almost 65% in the last 15 years. It is more frequent in regions with intense pesticide exposure [46]. In rural and underdeveloped countries, suicide by pesticide emerges as 30% of the world's suicides [22, 47].

After closely examining the victims who tested positive for medicines, our data showed that anticonvulsants/barbiturates/sedatives/hypnotics, antidepressants and benzodiazepines were the most frequently observed medicines in the toxicological analysis regardless of suicide method. These data suggest that this population was being treated for mental health conditions, as access to these medicines requires a prescription. Depression, substance use and psychosis constitute the most relevant risk factors, but anxiety, personality- and trauma-related disorders and organic mental disorders also significantly increase the suicide risk [45].

One limitation of this study is the lack of sociodemographic data in source documents, which would have enabled us to precisely match the suicide method to the psychiatric history and quantification analyses for toxicological findings. However, the underreporting and presence of unknown or unfilled variables are, unfortunately, common problems in sources used for public health and mortality research in Brazil [48]. Another limitation is that the toxicological analysis of a suicide case is not mandatory in the institution, as the forensic expert has the autonomy to request or not request any category of complementary exams. However, requesting a toxicological exam is a regular practice in the Legal Medicine Institute where this study was performed.

Our results suggest that strategies addressing known factors for suicide should include universal population strategies where the entire population should be reached, but primarily those who use over-the-counter drugs or have an illicit drug abuse history [6, 24,25]. Another possibility is to focus on mental health Programmes, which seem to be associated with lower hospitalizations for attempted suicide and psychiatric problems [49]. Investments in mental health in educational settings that aim to increase emotional competence and skills to deal with personal difficulties and stress should be helpful.

## Conclusion

This work involved a multidisciplinary forensic team composed of pathologists, anthropologists, toxicologists and police entities. The profiles of the suicide victims investigated in this study was predominantly composed of men with an average age of 40 years, mixed skin colour, with low educational level. The most frequently observed suicide method was hanging, followed by intoxication. Most victims presented positive toxicological results, especially for medicines, illicit drugs and pesticides. Considering the complexities involved with the suicide phenomenon, it is necessary to establish intersectoral strategies to reduce it.
